# [Corrigendum] Azurocidin 1 inhibits the aberrant proliferation of triple‑negative breast cancer through the regulation of pyroptosis

**DOI:** 10.3892/or.2024.8708

**Published:** 2024-01-24

**Authors:** Shanshan Lei, Shutong Li, Weiwei Xiao, Qiuping Jiang, Shifan Yan, Wen Xiao, Jiaodi Cai, Jingjing Wang, Lianhong Zou, Fang Chen, Yanjuan Liu, Yu Jiang

Oncol Rep 50: 188, 2023; DOI: 10.3892/or.2023.8625

Following the publication of the above article, the authors drew to our attention that they had made a couple of inadvertent errors in assembling Figs. 4 and 5; first, for the BT-549 cell line, the data shown for the Pro-caspase-1/Cleaved caspase-1 in Fig. 5 and the GSDMD-F/GSDMD-N data in Fig. 4B were identical, and had been derived from the same original source; secondly, in Fig. 4A, the data shown correctly for the GSDMD BT-549 cell line had also inadvertently been included in this figure to represent the MDA-MB-231 cell line.

The revised and corrected versions of Figs. 4 and 5, showing the correct western blotting data for the GSDMD experiment in Fig. 4A and the Pro-caspase-1/Cleaved caspase-1 data for the BT-549 cell line in Fig. 5, are shown in the next two pages. The authors regret that these errors in the assembly of Figs. 4 and 5 went unnoticed before the article was published, and thank the Editor of *Oncology Reports* for granting them the opportunity to publish this corrigendum. All the authors agree with the publication of this corrigendum; furthermore, they apologize to the readership of the journal for any inconvenience caused.

## Figures and Tables

**Figure 4.**
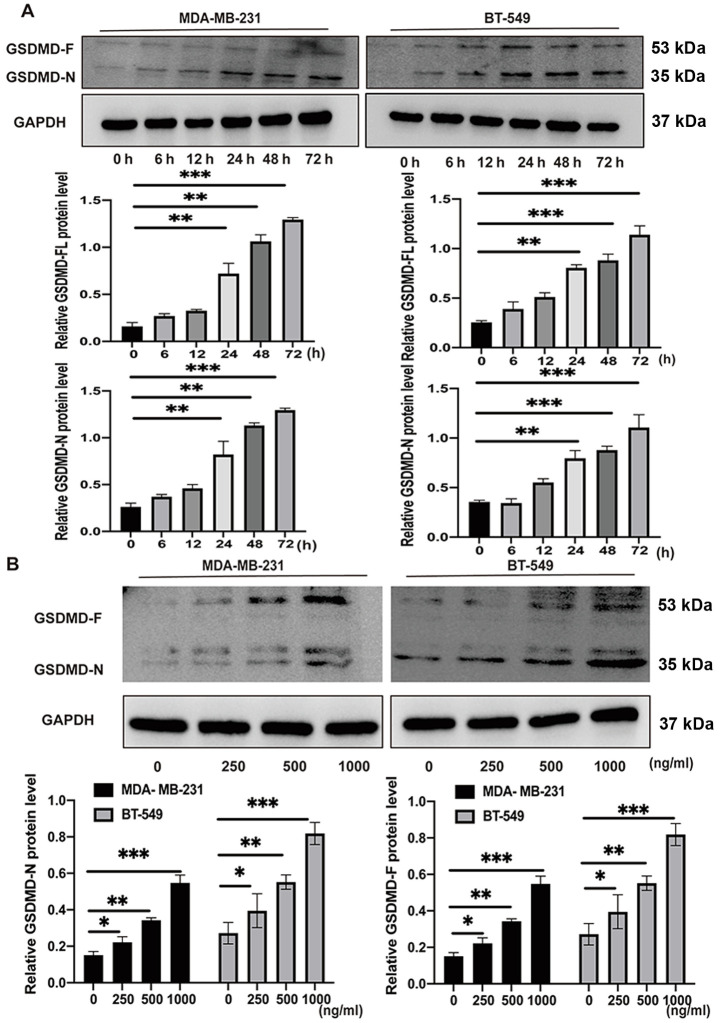
GSDMD protein level is detected in TNBC cell lines with exogenous AZU1 stimuli using western blot analysis. (A) GSDMD protein level in TNBC cell lines treated with 500 ng/ml AZU1 at different time points. **P<0.01 and ***P<0.001; (n=3). (B) GSDMD protein level in TNBC cell lines treated with different concentrations of AZU1. *P<0.05, **P<0.01 and ***P<0.001; (n=3). GSDMD, gasdermin D; TNBC, triple-negative breast cancer; AZU1, azurocidin 1.

**Figure 5.**
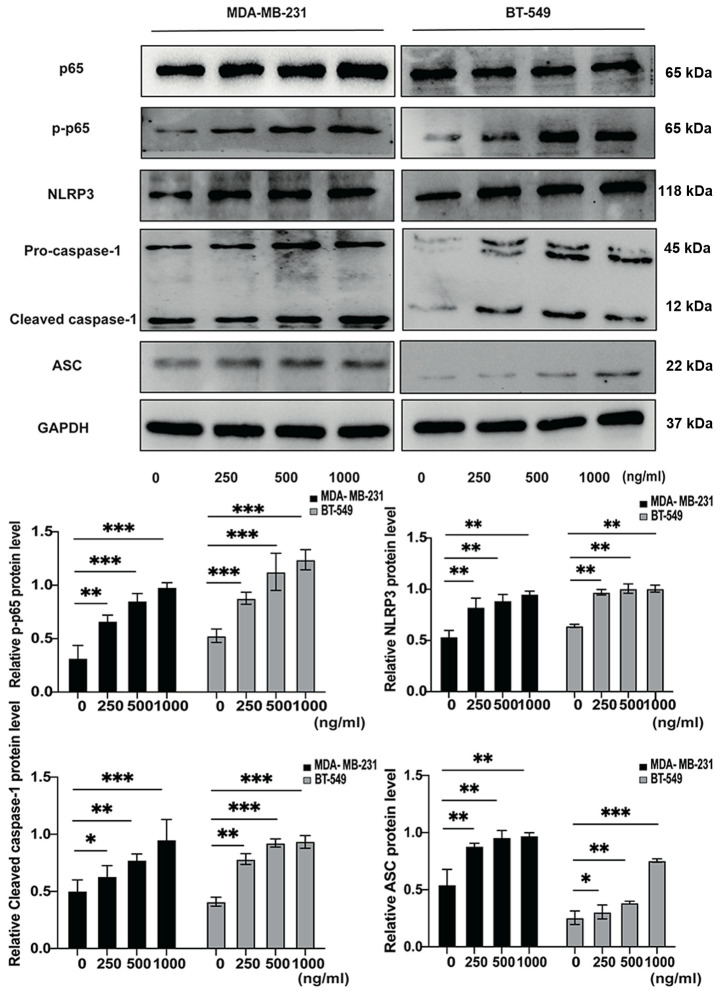
Western blot analysis is conducted to investigate the protein expression levels of p-p65, p65, NLRP3, pro-caspase-1, cleaved caspase-1 and ASC in TNBC cell lines with exogenous AZU1 stimuli. *P<0.05, **P<0.01 and ***P<0.001; (n=3). NLRP3, NLR family pyrin domain containing 3; ASC, apoptosis-associated speck-like protein containing a CARD; TNBC, triple-negative breast cancer; AZU1, azurocidin 1.

